# Social trust and subjective well-being of first-generation college students in China: the multiple mediation effects of self-compassion and social empathy

**DOI:** 10.3389/fpsyg.2023.1091193

**Published:** 2023-04-24

**Authors:** Qun Wang, Kuiyun Zhi, Baohua Yu, Jun Cheng

**Affiliations:** ^1^Institute of Educational Sciences, Huazhong University of Science and Technology, Wuhan, China; ^2^Mental Health Education and Counseling Center, Chongqing University, Chongqing, China; ^3^School of Public Policy and Administration, Chongqing University, Chongqing, China; ^4^Center for Social Security Studies, Wuhan University, Wuhan, China

**Keywords:** subjective well-being, social trust, self-compassion, social empathy, first-generation college students (FGCS), COVID-19, China

## Abstract

Previous studies have found that subjective well-being is associated with social trust, self-compassion, and social empathy. Based on online fieldwork with 662 first-generation college students (54.7% male) in China, this study aimed to investigate the serial mediation effects of self-compassion and social empathy on the relationship between social trust and subjective well-being. The results showed that subjective well-being was significantly positively correlated with social trust, trust in people, self-compassion, and social empathy. Both self-compassion and social empathy partially mediated the relationship between social trust and subjective well-being, and fully mediated the association between trust in people and subjective well-being. We used a serial mediation model to estimate the effect of general social trust, including trust in people, on subjective well-being. The findings that self-compassion and social empathy mediated the relationship between trusting attitudes toward society, especially people, and subjective well-being expand the literature on social trust and the mechanism of social trust on subjective well-being. The results also highlight the significance of improving mental health education and intervention among first-generation college students in China.

## 1. Introduction

First-generation college students are generally defined as those students whose parents have not attained college education or a college degree (Ishitani, 2006; Schwartz et al., 2018). Many scholars have examined the challenges faced by these students at university from a deficit perspective. They suggest that compared to non-first-generation college students, the lack of resources (e.g., financial, social-classical, cultural) of first-generation college students contributes to their difficulties at university, including academic experiences, cultural adaptation, and career management (see the literature review in Tian and Yu, 2021). However, other scholars have explored the psychosocial advantages of first-generation college students from an advantage perspective. Positive psychosocial forms of capital include mental resilience, self-efficacy, optimism, hope, responsibility, and a sense of purpose (e.g., Wright et al., 2013; Xie, 2016; Tian and Yu, 2021). These complex characteristics may be connected to these students’ mental health and well-being (e.g., Ellis et al., 2019; McCarron, 2022). At present, the number of first-generation college students in China accounts for 46.8–75% of all Chinese college students (Jiao, 2020). Some studies have suggested that these Chinese students are also disadvantaged at university, as is also the case for students in other countries (Tian and Yu, 2021; Fang and Lu, 2022). However, few researcher has focused on the positive psychosocial development of first-generation students in China, especially their mental health and well-being during the COVID-19 pandemic. Studies have found that the social life, mental health, life satisfaction and subjective well-being of college students around the world, including China, have been negatively affected by the COVID-19 pandemic since 2020 (Li et al., 2020; Wang and Zhao, 2020; Hagedorn et al., 2022). Thus, it would be useful to investigate the subjective well-being of first-generation college students in China.

The literature has concluded that the lack of family social and cultural capital negatively affects first-generation college students’ performance (e.g., academic, communication) at university (Soria and Stebleton, 2012; Lu and Hu, 2015; Fang and Lu, 2022), which may in turn reduce their subjective well-being index. Social capital theory (e.g., Putnam, 1993; Lin, 2001) holds that social capital is positively associated with people’s health and well-being. As the core element of social capital (e.g., Putnam, 1993), social trust moderates the negative effects of perceived COVID-19 stress on anxiety (Li and Lyu, 2021) and promotes people’s subjective well-being (e.g., Helliwell et al., 2014). Therefore, it seems reasonable to assume that social trust is positively related to subjective well-being among first-generation college students in China.

First-generation college students may experience difficulties with self-cognition and social interaction due to a shortage of family social and cultural capital, which might negatively affect their self-compassion and social empathy (Zhou and Bao, 2013; Lu and Hu, 2015; Fang and Lu, 2022). Healthy and nonjudgmental understanding of oneself is at the core of self-compassion (Neff, 2003a). When individuals have a high level of social trust, this contributes to their engagement in interaction, solidarity, and assistance (Kanovsky and Halamová, 2020), promotes empathy toward others (Bradshaw et al., 2019), and reduces serious conflicts and exclusionism among different groups in society (Thomsen et al., 2021). Moreover, in previous studies subjective well-being was positively related to self-compassion (e.g., Yang et al., 2016) and social empathy (e.g., Wei et al., 2011). These findings show that subjective well-being, social trust, self-compassion, and social empathy are closely associated.

In summary, few study has explored the relationship between trust in society and subjective well-being, as mediated through self-compassion and social empathy. Therefore, this study sought to investigate the serial mediation effects of self-compassion and social empathy on the association between social trust and subjective well-being, based on data from an online questionnaire.

## 2. Literature review and research hypothesis

### 2.1. Subjective well-being and social trust

Well-being is one of the ultimate goals of human life. Subjective well-being is defined as the subjective perception, evaluation, and satisfaction of quality of life (Campbell et al., 1976). Easterlin (1974) discussed the relationship between economic growth and income and well-being in 1974. The cross-sectional data showed that economic growth significantly impacted well-being, and that individuals’ income levels were positively related to well-being. Since then, different scholars have gradually discovered many complex factors that have an impact on subjective well-being. Alesina et al. (2004) divided these influencing factors of well-being into two categories: personality characteristics, including age, gender, ethnicity, education, health, and marital status; and social characteristics, including unemployment rate, income inequality, politics, and institutions. According to social capital theory (Putnam, 1993; Lin, 2001), social capital resources, which are embedded in various social networks at macro and micro levels and generated through interactions and relationships among people, can enhance people’s well-being. One study even concluded that social capital had stronger impact on subjective well-being and life satisfaction than other factors, including income (Bjørnskov, 2003). In addition to academic-related factors, some studies argued that psychosocial capital had significant effects on the subjective well-being of first-generation college students (Wang and Castañeda-Sound, 2011; Wright et al., 2013; McCarron, 2022). First-generation college students perform worse than other college students (e.g., in their academic performance) due to a lack of psychosocial capital, which has a detrimental impact on their life satisfaction and subjective well-being (Pascarella et al., 2004; Soria and Stebleton, 2012; Garriott, 2020).

Social trust is the core of social capital (e.g., Putnam, 1993). According to previous literature (e.g., Lin, 2001; Jing and Yang, 2013; Kye and Hwang, 2020; Li and Lyu, 2021), an individual’s attitudes of trust in people at the micro level and trust in organizations at the macro level constitute their overall trust attitudes for society. Trust in people, as the individual’s general trust attitude toward most people in society, usually does not mean specific trust in acquaintances (Peng, 2000; Uslaner, 2002; Iacono et al., 2021) and does not depend on people’s own interpersonal relationships (Lewis and Weigert, 1985). Trust in organizations refers to people’s general trust attitudes toward most organizations in society, including governmental and non-governmental organizations (e.g., Kye and Hwang, 2020). Social trust contributes short-term altruism and long-term self-interest (Putnam, 1993). Trust in people can positively predict an individual’s subjective well-being and life satisfaction (e.g., Helliwell et al., 2014). People who reside in cities with high levels of social trust have a higher level of well-being than those who reside in cities with low levels of social trust (Lu et al., 2020). Organizational trust (e.g., government, social groups, and enterprises) has been closely linked to well-being and life/job satisfaction (e.g., Hu and Xiao, 2021; Johannsen and Zak, 2021). Some studies on the relationship between social trust and subjective well-being are considered to suffer from the problems of potential endogeneity (Awaworyi Churchill and Mishra, 2017). However, Lu et al. (2020) confirmed a causal relationship between social trust and subjective well-being based on longitudinal evidence.

Many studies on the association between social trust and well-being have been conducted in developed countries, but there has been an insufficient focus on developing countries. A few scholars have begun to focus on this issue in China in recent years. Bai et al. (2019) concluded that Chinese trust in strangers was positively related to subjective well-being. A longitudinal study argued that social trust significantly positively predicted the subjective well-being of Chinese individuals, including college students (Lu et al., 2020). Additionally, some researchers suggested that trust in organizations (e.g., government and enterprise) was positively associated with Chinese subjective well-being (e.g., Qi and Lai, 2013; Hu and Xiao, 2021; Wang et al., 2021).

On the basis of the above findings, we formulated a hypothesis about the relationship between social trust and subjective well-being.

*H1*: Social trust, including trust in people and organizations, positively predicts subjective well-being among first-generation college students in China.

### 2.2. Mediator of self-compassion

Self-compassion is the ability to regard one’s feelings of suffering with a sense of warmth, connection, and concern (Neff, 2003a,b). Traditional compassion or pity focuses only on the suffering of others. However, Neff (2003b) argued that self-compassion is a healthy self-attitude, which emphasizes self-kindness, self-concern, and self-understanding. Self-compassion implies a positive self-concept and self-connection. People with self-compassion who encounter difficulties and setbacks can increase their positive emotions and the level of well-being through encouraging themselves to take positive actions (Neff, 2003a; Neff et al., 2008; Bluth et al., 2017). Although first-generation college students suffer from several disadvantages, their positive self-concept has been confirmed to profoundly impact their subjective well-being (Wang and Castañeda-Sound, 2011). Moreover, studies in different cultural contexts, such as China, Thailand, and the United States, demonstrated that self-compassion is positively related to subjective well-being and life satisfaction (Neff, 2003a; Neff et al., 2008; Yang et al., 2016). During the COVID-19 pandemic, self-compassion reduced individuals’ fear of the virus and enhanced their subjective well-being (Deniz, 2021).

Although the relationship between social trust and self-compassion has received little attention, some studies have concluded that trust is closely related to self-compassion. Crocker and Canevello (2008) found that interpersonal trust among adolescents was positively related to self-compassion, while a study of Chinese high school students concluded that self-compassion was positively associated with general trust (Yang et al., 2019). Social trust reflects an individual’s pro-sociality and goodwill toward society as a whole (Putnam, 1993; Crocker and Canevello, 2008), and includes people’ self-kindness and self-concern. Moreover, Kudesia and Reina (2019) concluded that interacting with trustworthy people could predict college students’ mindfulness, a core component of self-compassion (Neff, 2003a,b). Extending these findings to the association between social trust and self-compassion, we speculate that social trust contributes to healthy self-concern and self-kindness, as well as reducing negative emotions and enhancing subjective well-being.

On the basis of these evidences, we proposed a hypothesis regarding social trust, self-compassion, and subjective well-being.

*H2*: Self-compassion mediates the relationship between social trust and subjective well-being among first-generation college students in China.

### 2.3. Mediator of social empathy

“Social empathy is the ability to understand people by perceiving or experiencing their life situations and as a result gain insight into structural inequalities and disparities” (Segal, 2011, pp. 266–267). Traditionally, interpersonal empathy occurs between individuals or in small groups. However, Segal et al. (2017) claimed that based on a deep understanding of the context, social empathy emphasizes empathy for heterogeneous groups, especially vulnerable or minority groups. Empathy among college students is an altruistic behavior that also promotes other altruistic behaviors (Li et al., 2015; Segal et al., 2017). The empathy-altruism hypothesis holds that the core motivation and ultimate purpose of empathy is altruism, i.e., meeting other people’s needs and increasing the spiritual or material well-being of others (Batson et al., 2015). However, some scholars suggest that people have an emotional stake in promoting the welfare of others, i.e., increasing their own life satisfaction and happiness (de Waal, 2008). The faculty of College of New Jersey supported first-generation college students to gain empathy and understanding during the COVID-19 pandemic, helping them to succeed and improving the effectiveness and satisfaction of teaching (Chan et al., 2020). Previous studies have supported the positive association between social empathy and subjective well-being (Segal, 2012). Moreover, Wei et al. (2011) confirmed that the higher the empathy of college students, the higher the level of their subjective well-being.

Little attention has been given to the relationship between social trust and social empathy. Social capital theory holds that social trust implies social understanding and social support for others (Putnam, 1993). Social trust has been suggested to promote acceptance of minorities (see the literature review in Thomsen et al., 2021), which was consistent with the connotation of social empathy. Furthermore, Petrocchi et al. (2021) confirmed that college students’ trust beliefs in other people could predict their empathy. People’s trust within patient online communities is considered to positively influence their empathy (Zhao et al., 2013). Accordingly, these findings support the positive relationship between social trust and social empathy.

Extrapolating these findings to first-generation college students, social trust could promote their understanding and support for others, enable them to empathize with others, and ultimately enhance their level of life satisfaction and well-being. Thus, we proposed a relationship linking social trust, social empathy, and subjective well-being.

*H3*: Social empathy mediates the relationship between social trust and subjective well-being among first-generation college students in China.

### 2.4. Serial mediation effects of self-compassion and social empathy

Social capital theory (Putnam, 1993; Lin, 2001) holds that through social interaction and relations with others, people can gain tangible and intangible resources, i.e., social capital, at the individual, group, and organizational levels; and social capital brings material or spiritual benefits to individuals, groups, organizations, and even nations, including promoting well-being. These social relational networks should also include the connection and relationship with oneself. Self-compassion, as a sense of healthy self-concern, self-attitude, and self-connection (Neff, 2003b), has been closely associated with well-being (Yang et al., 2016; Bluth et al., 2017; Deniz, 2021), and should promote interpersonal interaction and concern, as well as empathy for others (Neff, 2003a). Furthermore, social empathy, built on individual empathy and applied empathy to social systems, emphasizes the deep understanding of different people and communities in society (Segal, 2011). It implies indirect and direct connections and social interaction between individuals and other people or organizations in society, which contribute to the well-being of individuals or the whole society (Wei et al., 2011; Segal, 2012). Thus, we proposed a serial two-mediator model exploring social trust, self-compassion, social empathy, and subjective well-being.

*H4*: Self-compassion and social empathy serially mediate the relationship between social trust (e.g., trust in people and organizations) and subjective well-being.

## 3. Materials and methods

### 3.1. Procedure and participants

In this study, an overall sampling method was used to select students from six colleges at a university in the city of Chongqing, China for participation in a questionnaire survey. The students were informed that their participation in a survey on college students’ attitudes toward social life was on a voluntary basis. The survey was conducted anonymously and the students gave their consent for the researchers to use the survey data for scientific research purposes. The researchers sent a link of the online questionnaire to the participants via a professional survey website^1^. All participating students were required to complete five questionnaires, including a basic personal information questionnaire, the Social Trust Questionnaire, the Social Empathy Index Scale, the Self-Compassion Scale (Short Version), and the Index of Subjective Well-being Scale.

We collected a total of 1,049 questionnaires. If only one parent had a bachelor’s degree or higher degree (*n* = 354), these data did not meet the requirements and were excluded from the analysis. Then we eliminated the invalid questionnaires (*n* = 33) that were completed too quickly and with invalid answers. Finally, we collected 662 valid surveys of first-generation college students. The participants included 362 male students (54.7%) and 300 female students (45.3%): 321 freshmen (48.5%), 169 sophomores (25.5%), 114 juniors (17.2%), and 58 seniors (8.8%). There were 344 students from rural areas (52%), 212 from towns (32%), and 106 from urban areas (16%). All participants were at least 18 years old.

The Harman one-way test was used to test for the presence of a common method bias effect. Factor analysis was performed on all questionnaire items and the results showed that the variance explained by the first principal component was 20.07% (<40%). Therefore, there were no significant common method bias effects among the variables in this study.

### 3.2. Measures

#### 3.2.1. Dependent variable

Subjective well-being was measured using the Index of Subjective Well-being Scale (Campbell et al., 1976), which contains nine items across two parts (see Supplementary Appendix): the index of general affect containing eight items (e.g., “What is the affective state you are experiencing now?”) and the life satisfactory questionnaire containing one item (i.e., “How satisfied are you with your whole life recently?”). A 7-point rating scale was applied, ranging from “strongly disagree (1)” to “strongly agree (7).” The weighted sum score of the two parts was used to examine the level of overall subjective well-being. A higher score indicates a higher level of subjective well-being. This scale is widely used in China (Shi et al., 2018), and the validity coefficient between the index of general affect and life satisfaction questionnaire was 0.55 (Wang et al., 1999). The Omega coefficient of the Index of Subjective Well-being Scale in this study was 0.919.

#### 3.2.2. Independent variable

Based on the existing literature (e.g., Ma, 2017; Lundberg and Abdelzadeh, 2019; Kye and Hwang, 2020; Iacono et al., 2021; Roychowdhury, 2021), eight questions were developed to measure social trust in this research (see Supplementary Appendix). Trust in people was assessed using six items (e.g., “In general, do you agree that most people can be trusted?”), and trust in organizations was assessed using two items (e.g., “In general, do you agree that most governmental organizations can be trusted?”). The questionnaire was scored on a 5-point Likert scale (1 = completely disagree, 5 = completely agree). We used the sum score of participants’ trust in people and organizations as overall social trust. The higher the score, the higher the level of social trust. In our research, the omega coefficient of overall social trust was 0.871.

#### 3.2.3. Mediator variables

One of the mediator variables in this study was self-compassion, which was measured with the Self-Compassion Scale-Short Form (Raes et al., 2011). The scale has 12 items across six sub-dimensions (see Supplementary Appendix): over-identification (two items, e.g., “When I’m feeling down I tend to obsess and fixate on everything that’s wrong.”), self-kindness (two items, e.g., “When I’m going through a very hard time, I give myself the caring and tenderness I need.”), mindfulness (two items, e.g., “When something painful happens, I try to take a balanced view of the situation.”), isolation (two items, e.g., “When I’m feeling down, I tend to feel like most other people are probably happier than I am.”), common humanity (two items, e.g., “I try to see my failings as part of the human condition.”), and self-judgment (two items, e.g., “I’m disapproving and judgmental about my own flaws and inadequacies.”). The form is scored on a 5-point rating scale (1 = almost never, 5 = almost always). We took the sum scores of all parts of the scale to represent the overall level of self-compassion. Higher scores indicate better self-compassion. This scale showed adequate internal consistency (Cronbach’s alpha ≥0.86) and a very good correlation with the long form of the Self-Compassion Scale (*r* ≥ 0.97) (Raes et al., 2011). The omega coefficient of the total scale in this study was 0.717.

Social empathy was another mediator variable in this study. It was measured with the Social Empathy Index Scale (Segal et al., 2017). This scale contains 40 items across seven parts (see Supplementary Appendix): affective response (five items, e.g., “When I see someone being publicly embarrassed I cringe a little.”), emotion regulation (four items, e.g., “Emotional stability describes me well.”), affective mentalizing (four items, e.g., “I am good at understanding other people’s emotions.”), perspective-taking (five items, e.g., “I can imagine what the character is feeling in a good movie.”), self-other awareness (four items, e.g., “I can tell the difference between my friend’s feelings and my own.”), contextual understanding of systemic barriers (nine items, e.g., “I believe adults who are in poverty deserve social assistance.”), and macro self-other awareness/perspective taking (nine items, e.g., “I confront discrimination when I see it.”). According to the actual situation in China, we revised some of the items, i.e., “different racial groups” and “in the United States’ educational system” were replaced with “different ethnic groups” and “in the Chinese educational system,” respectively. It is scored on a 6-point rating scale (1 = never, 6 = always). The sum score of seven parts of the scale was used to indicate the social empathy index. Higher scores indicate higher levels of social empathy. The Social Empathy Index Scale was widely used with college students (Segal, 2012), and the internal consistency of the scale was examined (Cronbach’s alpha ≥0.86) (Wagaman et al., 2018). The omega coefficient of this scale in this study was 0.927.

#### 3.2.4. Control variables

Considering the possible important effects of demographic variables, we also controlled for other demographic variables (e.g., gender, grade, and district) to reduce omitted variable bias. The existing literature confirmed that demographic factors, especially gender, were likely to be associated with social trust and subjective well-being (e.g., Bai et al., 2019; Lu et al., 2020). Lu et al. (2020) suggested that the well-being of male and urban residents was more easily influenced by social trust in China, compared with the well-being of female and rural residents. Moreover, some scholars have reported demographic differences among first-generation college students (e.g., gender, grade, and district; review in Jiao, 2020), with some Chinese scholars controlling for demographic variables (e.g., gender) in their studies (Xie, 2016; Jiao, 2020). Therefore, we included gender, grade, and domicile district in the model as control variables, all coded as dummy variables. Gender was coded as 1 = male and 2 = female. Grade was coded as 1 = freshman, 2 = sophomore, 3 = junior, and 4 = senior. Domicile district was coded as 1 = participants from rural areas, 2 = participants who lived in towns, and 3 = participants from urban areas.

### 3.3. Statistical analysis

We used SPSS 27.0 and AMOS 24.0 to conduct the statistical analysis. First, SPSS was used to conduct descriptive statistics and correlational analysis. Then, AMOS 24.0 was used to conduct a path analysis of the tested model with the maximum likelihood estimation. In addition, the bootstrap method with a sample size of 5,000 was used to test the mediation effects of self-compassion and social empathy on the relationship between social trust and subjective well-being, and the path coefficient of the mediation effect was calculated and reported.

## 4. Results

### 4.1. Descriptive statistics and correlation analysis

Table 1 shows the descriptive statistics and correlation coefficients between the variables. The results of the correlation analysis show that the correlation among all of the variables is significantly positive (*p* < 0.001). It indicated that subjective well-being was significantly correlated with overall social trust, trust in people, trust in organizations, self-compassion, and social empathy. The results indicated that the mean value of trust in organizations was greater than the mean value of trust in people (*t* = 27.447, *df* = 661, *p* < 0.001, Cohen’s *d* = 0.515) among the first-generation college students in China.

**Table 1:** Descriptive statistics and correlations among the variables.

Variable	Mean	*SD*	1	2	3	4	5
1. Social trust	24.24	3.89	1				
2. Trust in people	6.89	1.29	0.981^***^	1			
3. Trust in organizations	24.24	4.87	0.813^***^	0.684^***^	1		
4. Self-compassion	159.35	24.99	0.161^***^	0.162^***^	0.117^***^	1	
5. Social empathy	39.46	4.83	0.207^***^	0.201^***^	0.178^***^	0.239^***^	1
6. Subjective well-being	41.62	8.76	0.238^***^	0.229^***^	0.208^***^	0.327^***^	0.372^***^

***p < 0.001.

### 4.2. Serial mediation effects of social trust and subjective well-being

**Figure 1:**
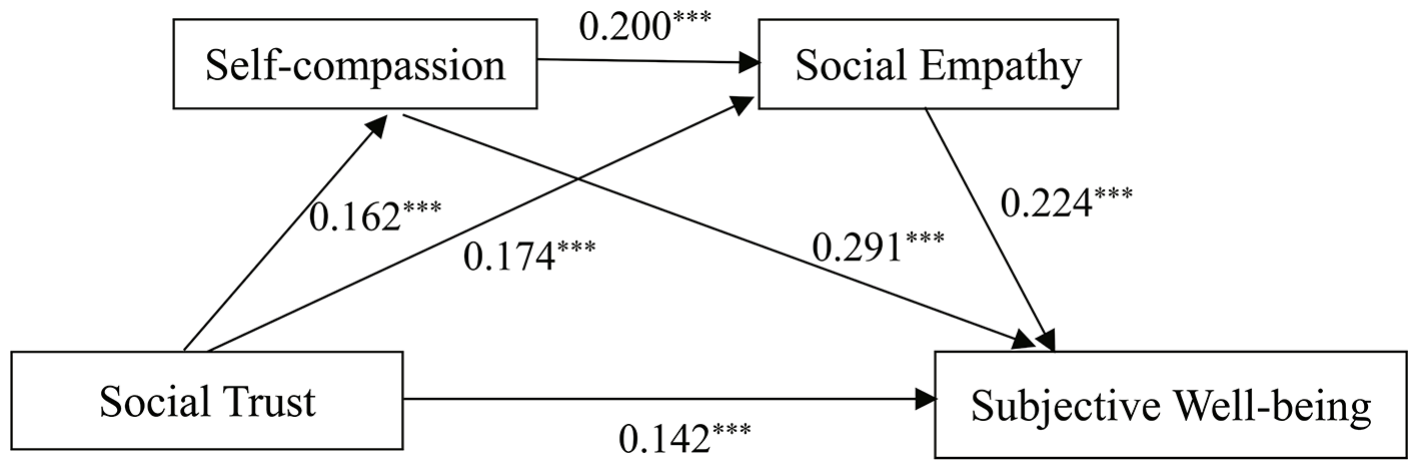
Path analysis of social trust, self-compassion, social empathy, and subjective well-being. ***p < 0.001.

The overall fit of the tested model was analyzed using AMOS 24.0. The results showed acceptable model fit indices (*χ^2^*/*df* = 1.49, CFI = 0.988, TLI = 0.960, SRMR = 0.024, and RMSEA = 0.027). We included gender, grade, and domicile district as control variables, and used the bootstrap method (*n* = 5,000) to test the serial mediation effects of social trust on subjective well-being. Figure 1 shows the results of the path analysis, while Table 2 shows the effect values of the indirect and direct pathways. The results demonstrated that social trust had a significant positive direct effect on subjective well-being (*β* = 0.142, *p* < 0.001). Hypothesis 1, that overall social trust positively predicts subjective well-being, was verified.

**Table 2:** Analysis of the mediation effects of self-compassion and social empathy.

	*β*	SE	95% *CI*		Relative Mediating Effect
			Lower	Upper	
Direct effect	0.142	0.039	0.065	0.215	60.43%
Total indirect effect	0.093	0.019	0.058	0.133	39.57%
Indirect effect 1: ST➔SC➔SWB	0.047	0.013	0.024	0.076	20.00%
Indirect effect 2: ST➔SE➔SWB	0.039	0.018	0.018	0.066	16.59%
Indirect effect 3: ST➔SC➔SE➔SWB	0.007	0.003	0.003	0.014	2.98%

ST, social trust; SC, self-compassion; SE, social empathy; SWB, subjective well-being.

The path coefficient between social trust and self-compassion was 0.162 (*p* < 0.001), indicating that social trust significantly positively predicted self-compassion. The path coefficient between self-compassion and subjective well-being was 0.291 (*p* < 0.001), showing that self-compassion significantly partially mediated the association between social trust and subjective well-being (*β* = 0.047, *p* < 0.001, 95% CI: 0.024, 0.076). Additionally, the mediation effect of self-compassion explained 20% of the total effect. Hypothesis 2 was supported.

The path coefficient between social trust and social empathy was 0.174 (*p* < 0.001), and the path coefficient between social empathy and subjective well-being was 0.224 (*p* < 0.001). This indicates that social empathy partially mediated the relationship between social trust and subjective well-being (*β* = 0.039, *p* < 0.001, 95% CI: 0.018, 0.066). The mediation effect of social empathy explained 16.59% of the total effect. Hypothesis 3 of this study was supported.

The path coefficient between self-compassion and social empathy was 0.200 (*p* < 0.001), indicating that self-compassion was closely associated with social empathy. These results revealed the serial mediation effects of self-compassion and social empathy between social trust and subjective well-being (*β* = 0.007, *p* < 0.001, 95% CI: 0.003, 0.014). The serial mediation effects explained 2.98% of the total effect. Hypothesis 4 of this study was supported.

**Figure 2:**
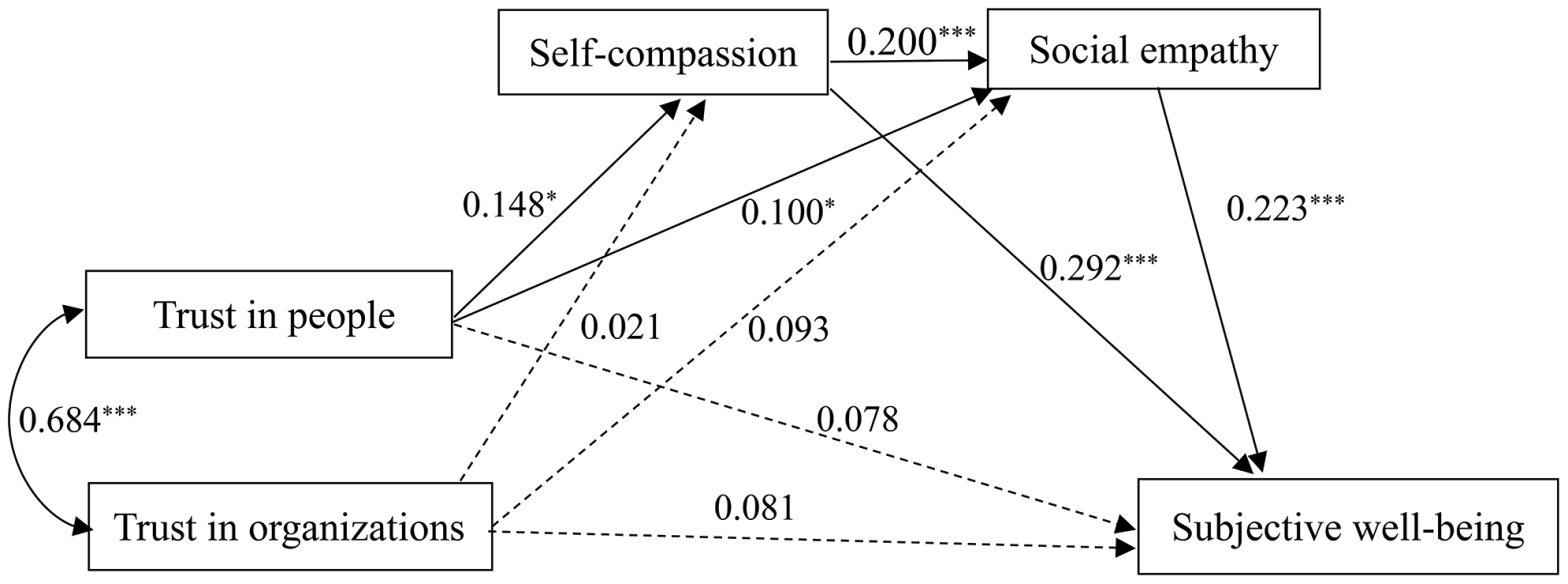
Path analysis of social trust in people and organizations, self-compassion, social empathy, and subjective well-being. ***p < 0.001, *p ≤ 0.05.

Furthermore, we examined the serial mediation effects of self-compassion and social empathy on the association between trust in people and organizations and subjective well-being. Figure 2 shows the path analysis. The results presented that trust in people did not have a significant direct effect on subjective well-being (*β* = 0.078, *p >* 0.05). The path coefficient between trust in people and self-compassion was 0.148 (*p* < 0.05), while the path coefficient between self-compassion and subjective well-being was 0.292 (*p* < 0.001). In addition, trust in people had a marginally significant effect on social empathy (*β* = 0.100, *p* ≤ 0.05), and had a significant effect on subjective well-being (*β* = 0.223, *p* < 0.001).

Considering that the direct effect of trust in people on subjective well-being was not statistically significant, self-compassion and social empathy played fully mediating roles in the relationship between trust in people and subjective well-being (*β* = 0.043, *p* < 0.05, 95% *CI*: 0.011, 0.081; *β* = 0.022, *p* < 0.05, 95% *CI*: 0.001, 0.050, respectively). Moreover, the path coefficient between self-compassion and social empathy was 0.200 (*p* < 0.001). This revealed that self-compassion and social empathy had serial mediation effects on the relationship between trust in people and subjective well-being (*β* = 0.007, *p* < 0.01, 95% CI: 0.002, 0.015). Moreover, compared to trust in people, trust in organizations did not significantly predict self-compassion (*β* = 0.021, *p >* 0.05), social empathy (*β* = 0.093, *p >* 0.05), and subjective well-being (*β* = 0.081, *p >* 0.05).

## 5. Discussion

The results indicated that both overall social trust and trust in people positively predicted well-being, which is consistent with previous studies (e.g., Helliwell et al., 2014; Awaworyi Churchill and Mishra, 2017; Bai et al., 2019). The results showed that trust attitudes toward the society are a positive psychological resource for Chinese first-generation college students, and that trusting attitudes toward society could promote the students’ well-being level (McCarron, 2022). The results of this study also illustrated that organizational trust did not significantly affect self-compassion, social empathy, and subjective well-being, which is inconsistent with the findings of previous studies (Awaworyi Churchill and Mishra, 2017; Hu and Xiao, 2021; Johannsen and Zak, 2021). Based on the interviews with first-generation college students, we found that their interactions with organizations were often characterized by positive interactions with certain special people, including their teachers, classmates, and even strangers who helped them. Giddens (1990) argued that individuals’ trust in a social system was related to their trust attitudes toward specific persons. Therefore, we further speculate that, compared to the effects of trust in organizations, Chinese first-generation college students’ subjective well-being and mental health are more significantly positively affected by trust beliefs in people.

The results showed that self-compassion partly mediated the relationship between social trust and subjective well-being, and fully mediated the relationship between trust in people and subjective well-being. Previous studies have demonstrated that self-compassion is closely associated with subjective well-being (Yang et al., 2016; Deniz, 2021). Trust in other people can enhance the students’ healthy self-conception and self-compassion (Kudesia and Reina, 2019). We contend that first-generation college students developed positive psychosocial capital (e.g., general trust attitude) to overcome the various difficulties in university and improve the level of positive self-compassion and well-being.

The results indicated that social empathy was the partial mediator between social trust and subjective well-being, and fully mediated the association between trust in people and subjective well-being. The results were consistent with past studies (Wei et al., 2011; Segal, 2012; Zhao et al., 2013; Petrocchi et al., 2021). Some scholars have confirmed that social empathy is closely associated with subjective well-being (Wei et al., 2011; Segal, 2012). Social trust, especially trust in people, can positively affect empathy for others (Zhao et al., 2013; Petrocchi et al., 2021), and promote acceptance of minorities (Thomsen et al., 2021). General trust attitudes among first-generation college students indirectly promote the level of their life satisfaction and well-being by improving the understanding, empathy and concern for other people and groups in society. The results of this study accord with but surpass the empathy-altruism hypothesis. Chinese first-generation college students, holding positive attitudes of social trust, have an emotional stake in understanding and empathy for society, thereby indirectly promoting their level of well-being.

The results of this study also indicated that self-compassion and social empathy have serial mediation effects between overall social trust, trust in people, and subjective well-being. The results were consistent with the past study (Neff, 2003a,b). Self-compassion emphasizes seeing one’s own experience in light of the common human experience and entails compassion for all people, including oneself and others in society (Neff, 2003a,b). Previous studies suggested that self-compassion could promote feelings of concern and empathy for others (Barnard and Curry, 2011) and foster a sense of social connectedness and responding to others (Neff, 2003a,b). Compared to the situations of other students, the psychosocial and family capital of first-generation college students is more likely to be a disadvantage. However, their positive self-concept, self-care, and self-compassion contribute to the care, understanding, empathy, and support for other people or groups in society, and to improving the level of mental health and subjective well-being. This result is consistent with the viewpoint of social capital theory, which suggests that social capital could bring material or spiritual benefits to individuals, organizations, and even society as a whole. Therefore, enhancing positive trust attitudes toward society and positive self-attitudes among first-generation college students is important in promoting their well-being and empathic interactions with others.

In conclusion, this study has several valuable theoretical and practical implications. We found the serial mediating effects of self-compassion and social empathy on the relationship between trust in society, including in people, and subjective well-being. These findings enrich the mechanism of social trust on subjective well-being, and expand the literature on social trust by developing a model of the association between social trust, self-compassion, and social empathy. It is noted that this study found a stronger effect of trust in people on subjective well-being, rather than trust in organizations; and self-compassion fully mediated the relationship between trust in people and subjective well-being. Therefore, this study used a serial mediation model to examine the effect of common trust beliefs in society, especially in people, on the subjective well-being of first-generation college students in China.

Furthermore, the findings in this study also have valuable implications for university educators and first-generation college students. Helping these students to improve the level of well-being and satisfaction, including in their lives and academic performance, contributes to their mental health. For mental health teachers and other educators, a more focused measure should be developed to enhance the psychosocial capital of first-generation college students. Appropriate interventions may include education on trust in society, self-compassion education, and empathy training. In particular, it is important to help first-generation college students to develop a positive healthy self-concept and learn to be concerned and care for themselves. Therefore, our findings that self-compassion and social empathy fully or partially mediate the association between trust attitudes toward society, especially people, and subjective well-being have implications for the mental health education and interventions among first-generation college students in China.

The present study also has several limitations. First, the participants contained a large number of freshmen and a small number of seniors. Due to the survey being carried out not long before senior students’ graduation in a university, many students were too busy to participate in the survey. Therefore, future studies should balance the samples across grades and different universities. Second, we acknowledge that the data from this cross-sectional study could not examine the causal relationships between variables. Future longitudinal studies need to further validate the results of this study. Third, the results of this study were based on the sample of first-generation students in China. It is meaningful to compare Chinese first-generation college students and second-generation college students and analyze the differences in the later research.

## Data availability statement

The raw data supporting the conclusions of this article will be made available by the authors, without undue reservation.

## Ethics statement

Ethical review and approval was not required for the study on human participants in accordance with the local legislation and institutional requirements. The patients/participants provided their written informed consent to participate in this study.

## Author contributions

QW: conceptualization, methodology, and writing a draft of the manuscript. KZ: conceptualization, revise and perfect the thesis, and supervising. BY: reviewing, editing, and supervising. JC: translation and complete manuscript writing. All authors contributed to the article and approved the submitted version.

## Funding

This study was supported by the Fundamental Research Funds from the Central Universities (Grant Nos. 2022CDJSKPY14 and 2018CDJSK01PT05), and the National Social Science Foundation Research Program of China (Grant No. 21BSH117).

## Conflict of interest

The authors declare that the research was conducted in the absence of any commercial or financial relationships that could be construed as a potential conflict of interest.

## Publisher’s note

All claims expressed in this article are solely those of the authors and do not necessarily represent those of their affiliated organizations, or those of the publisher, the editors and the reviewers. Any product that may be evaluated in this article, or claim that may be made by its manufacturer, is not guaranteed or endorsed by the publisher.

## Footnotes

^1^ https://www.wjx.cn
