# Supplementary material for: Social trust and subjective well-being of first-generation college students in China: the multiple mediation effects of self-compassion and social empathy
Source: Front Psychol. 2023 Apr 24;14:1091193. doi: 10.3389/fpsyg.2023.1091193 (PMC10164941; doi:10.3389/fpsyg.2023.1091193)
Supplement: Supplementary file 1 [file Table_1.pdf]

Appendix

Constructs	Items	
Subjective well-being (Campbell et al., 1976; Wang et al., 1999)	IGA1	What is the affective state you are experiencing now? Boring -- Interesting
	IGA2	What is the affective state you are experiencing now? (R) Enjoyable -- Miserable
	IGA3	What is the affective state you are experiencing now? Useless -- Worthwhile
	IGA4	What is the affective state you are experiencing now? (R) Friendly-- Lonely
	IGA5	What is the affective state you are experiencing now? (R) Full -- Empty
	IGA6	What is the affective state you are experiencing now? Discouraging -- Hopeful
	IGA7	What is the affective state you are experiencing now? Disappointing -- Rewarding
	IGA8	What is the affective state you are experiencing now? (R) Bring out the best in me -- Doesn't give me much change
	LS1	How satisfied are you with your whole life recently?
Social Trust (e.g., Iacono et al., 2021; Kye and Hwang, 2020)	TP1	In general, do you agree that most people can be trusted?
	TP2	In general, do you agree that it is important to try to be careful when dealing with most people in society? (R)
	TP3	In general, do you agree that most people are fair and honest?
	TP4	In general, do you agree that most people always want to take advantage of others? (R)
	TP5	In general, do you agree that most people try to do their best to help others?
	TP6	In general, do you agree that most people only think of themselves most of the time? (R)
	TO1	In general, do you agree that most governmental organizations can be trusted?
	TO2	In general, do you agree that most non-governmental organizations can be trusted?
Self-compassion (Raes et al., 2011)	OI1	When I fail at something important to me I become consumed by feelings of inadequacy. (R)
	OI2	When I'm feeling down, I tend to obsess and fixate on everything that's wrong. (R)
	SK1	I try to be understanding and patient towards those aspects of my personality I don't like.

	SK2	When I'm going through a very hard time, I give myself the caring and tenderness I need.
	M1	When something painful happens I try to take a balanced view of the situation.
	M2	When something upsets me I try to keep my emotions in balance.
	I1	When I'm feeling down, I tend to feel like most other people are probably happier than I am. (R)
	I2	When I fail at something that's important to me, I tend to feel alone in my failure. (R)
	CH1	I try to see my failings as part of the human condition.
	CH2	When I feel inadequate in some way, I try to remind myself that feelings of inadequacy are shared by most people.
	SJ1	I'm disapproving and judgmental about my own flaws and inadequacies. (R)
	SJ2	I'm intolerant and impatient towards those aspects of my personality I don't like. (R)
Social empathy (Segal et al., 2017)	AR1	When I see someone receive a gift that makes them happy, I feel happy myself.
	AR2	When I see someone being publicly embarrassed I cringe a little.
	AR3	When I see someone accidentally hit his or her thumb with a hammer, I feel a flash of pain myself.
	AR4	When I am with someone who gets sad news, I feel sad for a moment too.
	AR5	Hearing laughter makes me smile.
	ER1	Emotional stability describes me well.
	ER2	When I get angry, I need a lot of time to get over it. (R)
	ER3	Friends view me as a moody person. (R)
	ER4	When I am upset or unhappy, I get over it quickly.
	AM1	I am good at understanding other people's emotions.
	AM2	When I see a person experiencing a strong emotion I can accurately assess what that person is feeling.
	AM3	When I see a person experiencing a strong emotion, I can describe what the person is feeling to someone else.
	AM4	I am aware of other people's emotions.
	PT1	I can consider my point of view and another person's point of view at the same time.
	PT2	I can imagine what the character is feeling in a good movie.
	PT3	I can imagine what it's like to be in someone else's shoes.
	PT4	I consider other people's points of view in discussions.
	PT5	I can agree to disagree with other people.

SOA1	I can tell the difference between someone else's feelings and my own.
SOA2	I can tell the difference between my friend's feelings and my own.
SOA3	I can explain to others how I am feeling.
SOA4	I am aware of what other people think of me.
CU1	I believe adults who are in poverty deserve social assistance.
CU2	I think the government needs to be a part of leveling the playing field for people from different ethnic groups.
CU3	I believe that people who face discrimination have added stress that negatively impacts their lives.
CU4	I believe government should protect the rights of minorities.
CU5	I believe people born into poverty have more barriers to achieving economic well-being than people who were not born into poverty.
CU6	I think it is the right of all citizens to have their basic needs met.
CU7	I believe the role of government is to act as a referee, making decisions that promote the quality of life and well-being of the people.
CU8	I believe that by working together, people can change society to be more just and fair for everyone.
CU9	I believe there are barriers in the Chinese educational system that prevent some groups of people from having economic success.
MSP1	I confront discrimination when I see it.
MSP2	I believe it is necessary to participate in community service.
MSP3	I am comfortable helping a person of a different race or ethnicity than my own.
MSP4	I take action to help others even if it does not personally benefit me.
MSP5	I can best understand people who are different from me by learning from them directly.
MSP6	I believe that each of us should participate in political activities.
MSP7	I feel it is important to understand the political perspectives of people I don't agree with.
MSP8	I have an interest in understanding why people cannot meet their basic needs financially.
MSP9	I believe my actions will affect future generations.

"R" means this item was reverse-scored. IGA: Index of General Affect; LS: Life Satisfactory; TP: Trust in people; TO: Trust in organizations; OI: Over-Identification; SK:

Self-Kindness; M: Mindfulness; I: Isolation; CH: Common Humanity; SJ: Self-Judgment; AR: Affective Response; ER: Emotion Regulation; AF: Affective Mentalizing; PT: Perspective-Taking; SOA: Self-Other Awareness; CU: Contextual Understanding of Systemic Barriers; MSP: Macro Self-other awareness/Perspective Taking.
